# Embodied time and the out-of-body experience of the self

**DOI:** 10.7717/peerj.8565

**Published:** 2020-03-13

**Authors:** Sylvie Droit-Volet, Sophie Monceau, Michaël Dambrun, Natalia Martinelli

**Affiliations:** Université Clermont Auvergne, Laboratoire de Psychologie Sociale et Cognitive, CNRS, UMR 6024, Clermont-Ferrand, France

**Keywords:** Timing, Time, Sense of time, Embodiment, Out-of-body, Body, Self-awareness, Self, Consciousness, Emotion

## Abstract

Using an out-of-body paradigm, the present study provided further empirical evidence for the theory of embodied time by suggesting that the body-self plays a key role in time judgments. Looking through virtual reality glasses, the participants saw the arm of a mannequin instead of their own arm. They had to judge the duration of the interval between two (perceived) touches applied to the mannequin’s body after a series of strokes had been viewed being made to the mannequin and tactile strokes had been administered to the participants themselves. These strokes were administered either synchronously or asynchronously. During the interval, a pleasant (touch with a soft paintbrush) or an unpleasant stimulation (touch with a pointed knife) was applied to the mannequin. The results showed that the participants felt the perceived tactile stimulations in their own bodies more strongly after the synchronous than the asynchronous stroking condition, a finding which is consistent with the out-of-body illusion. In addition, the interval duration was judged longer in the synchronous than in the asynchronous condition. This time distortion increased the greater the individual out-of-body experience was. Our results therefore highlight the importance of the awareness of the body-self in the processing of time, i.e., the significance of embodied time.

## Introduction

There is now a lot of evidence suggesting that estimated time is elastic. Indeed, judgments of time fluctuate according to our internal states (emotion, body movement, temperature). For example, the presentation duration of a stimulus is judged longer with a threatening stimulus that induces an emotion of fear than with a neutral stimulus (for a review, Droit-Volet, 2019). This lengthening of time estimates is thought to result from the experience of an acceleration of internal time (internal clock) in comparison to the conventional representation of a constant flow of external time. The judgment of time would thus derive from the conscious experience of internal time, which depends on the physiological state of the body (emotional state, movement) which is, in turn, obviously related to the activity of the nervous system, e.g., the transient activation of dopaminergic neurons that speeds up time estimates (Ogden et al., 2019; Simen & Matthew, 2016; Soares, Atallah & Paton, 2016).

To account for this sensitivity of time judgments to internal states, some authors have suggested the existence of what they call grounded or embodied time (Droit-Volet & Gil, 2009; Droit-Volet, 2014; Wittmann, 2014a; Wittmann, 2014b). Based on the theory of embodied cognition (Barsalou, 2008; Niedenthal, 2007), they argue that the judgment of durations is grounded in multiple ways, including through motor simulations, situated action, and bodily states. Bodily-self awareness would therefore contribute to time judgments, or at least explicit time judgments. Various types of evidence in favor of this idea can be found in the literature. Some studies of patients suffering from schizophrenia have suggested a relationship between their disorder of the bodily self and their difficulties in predicting events in time (Giersch & Mishara, 2017; Giersch, Lalanne & Isope, 2016; Martin et al., 2017). Their low sensitivity to short temporal asynchrony, which alters the perception of the continuity of the self in time, would explain the disturbance of the minimal self in schizophrenia (Lalanne, Van Assche & Giersch, 2012). Some studies have shown that the practice of mindfulness meditation, which focuses attention on the body (e.g., body scan meditation), modifies not only the awareness of body-self and of the present moment but also the judgment of durations (e.g., Dambrun, 2016; Dambrun et al., 2019; Droit-Volet et al., 2019; Droit-Volet & Dambrun, 2019; Wittmann, 2015; Wittmann, 2018). Other studies have highlighted the critical role of sensory-motor experience (embodied simulation) in the time judgment of emotional facial expressions (Droit-Volet, Brunot & Niedenthal, 2004; Effron et al., 2006; Fernandez & Garcia-Marques, 2019; Mondillon et al., 2007) or bodily expressions (e.g., Nather & Bueno, 2012; Nather et al., 2011; Vatakis et al., 2014; Droit-Volet & Gil, 2016). For example, Effron et al. (2006) showed that the temporal overestimation of facial expression of anger disappeared when the automatic motor mimicry of this facial expression was prevented by asking the participants to hold a pen in their mouths, i.e., when the participants did not themselves have the sensory-motor experience of the emotion observed in the other. Other studies have also shown that the participants with high awareness of body signals (heartbeat perception score) were more accurate in their temporal reproduction (Meissner & Wittmann, 2011). Although these different studies provide arguments in favor of embodied time, the idea still lacks adequate or sufficiently convincing support (Droit-Volet, 2018). The aim of the current study was to try to provide new empirical evidence by investigating whether time judgments change when the self is dissociated from the physical body, i.e., in the out-of-body experience. The present study is thus the first one to use the experimental paradigm of the out-of-body illusion to test time judgments.

The out-of-body experience is the feeling that the self is located outside of one’s own physical body. Self-awareness in our body results from the conjunction of multiple forms of information (Ehrsson, 2007; Guterstam et al., 2019). We are aware that our arm belongs to us, because when we move it to take an object, we receive proprioceptive information during the movement and we simultaneously see our arm moving towards the object. The out-of-body illusion therefore results from a match between the visual and proprioceptive information. In the experimental paradigm of the out-of-body illusion, participants look through virtual reality glasses at a body part (rubber arm) or the whole body of a mannequin, instead of at their own body (Botvinick & Cohen, 1998; Ehrsson, 2007). When they look down, they therefore do not see their arm but the mannequin’s arm (artificial body). They then see the experimenter stroking the mannequin with a small pen and simultaneously feel this tactile stimulation on their own body. The experimenter strokes the participant’s body (proprioception information) in synchrony with the stroke visually perceived on the artificial body (synchrony condition). In a control condition, the participants see and feel the stimulation alternately. The experimenter strokes the participants after they have seen the stroke administered to the artificial body (asynchrony condition).

The results of studies on the out-of-body illusion have demonstrated that participants experience the mannequin’s body as belonging to them more in the synchronous than in the asynchronous condition. In the synchronous condition, they clearly reported that they had the impression that the artificial body was their own body and that they felt the sensation of the touch of the paintbrush on the mannequin (Botvinick & Cohen, 1998; Ehrsson, 2007; Schmalzl & Ehrsson, 2011; Petkova & Ehrsson, 2001). Consequently, when participants feel the artificial body as their own, we can argue that the self is located outside the body, i.e., in the artificial body. In the out-of-body illusion, the participants thus feel the tactile stimulation administered to the mannequin in the absence of any direct proprioceptive stimulation of their own bodies.

Our hypothesis is that if self-awareness and the associated sensory-motor sensation represent critical factors for time judgments, the perception of an interval duration between two tactile stimulations given to the mannequin should be different after a synchronous (embodied time) and an asynchronous stimulation (perceived time) condition. As the studies on timing have systematically found that the perception of bodily or facial expressions associated with highly arousing emotions produces a temporal lengthening effect caused by the increase in the speed of the perceiver’s internal clock, we suggest that embodied time in the synchronous condition should also provide an overestimation of time. In the current study based on the out-of-body paradigm, the participants therefore had to judge the duration of the interval between two touches administered to the mannequin after a series of synchronous or asynchronous visual-tactile stimulations. In addition, a pleasant or an unpleasant touch was given to the mannequin during the inter-touch interval. The pleasant touch was administered with a soft paintbrush and the unpleasant one with a sharp knife. After making their temporal judgment, the participants also reported on a verbal scale the extent to which they could feel the touch given to the mannequin in their own bodies.

## Method

### Participants

Forty-seven first-year psychology students participated in this experiment in exchange for course credits (mean age = 20.1 years, SD = 1.45). They gave their written consent on a form which contained a simplified description of the procedure, as they were unaware of the specific aim of the experiment. The procedure was approved by the Research Ethics Committee of the university of Clermont-Auvergne (IRB00011540-2019-12).

### Material

**Figure 1:**
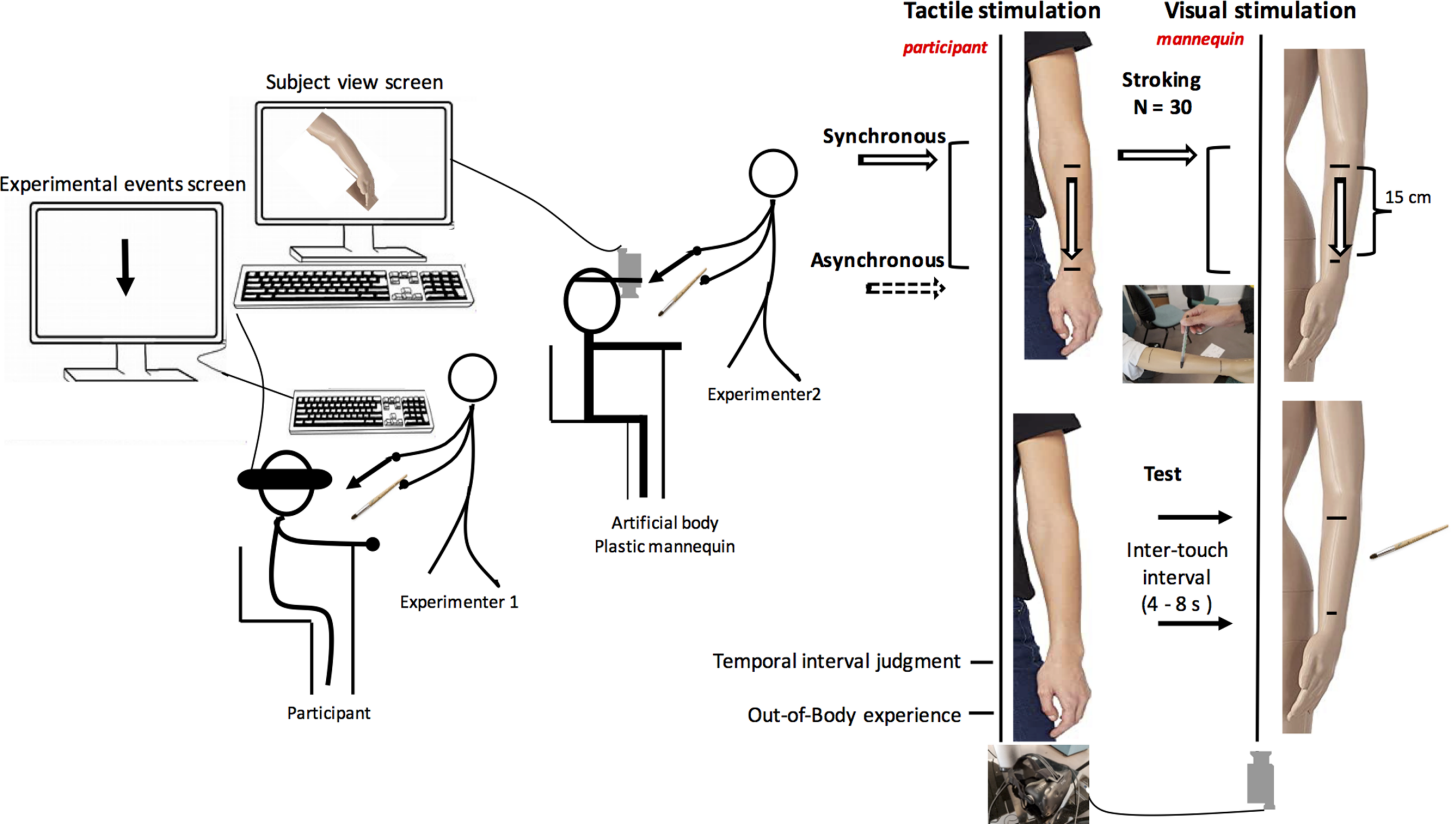
Experimental design.

The participants sat on a chair with their right arm resting on a chair back (Fig. 1). Beside them, there was a female plastic mannequin in the same position, dressed in a white T-shirt. On the arms of both the participant and the mannequin, there were two marks at a distance of 15 cm localizing the tactile stimuli used for the onset and the offset, respectively, of the interval duration to be judged. These marks were also used to localize the repeated stroking in the synchronous and asynchronous conditions. The strokes were applied from the first to the second mark. The tactile stimulus used for the temporal interval and the stroking stimulation was a small pen (1 cm diameter and 12 cm long). A first experimenter used this pen on the participant and a second one on the mannequin (Fig. 1). In the test trials described in the procedure, this latter experimenter also used a soft paintbrush for the pleasant stimulus and a threatening item (sharp knife) for the unpleasant stimulus.

Throughout the entire experiment, the participants wore virtual reality glasses (HTC Vive Steam VR) that were connected to a camera (Ricoh Theta V) attached to the mannequin’s head. The camera filmed the mannequin’s arm and sent the images live to the VR glasses. Therefore, when the participants looked toward their own arm through the VR glasses, they saw the mannequin’s arm instead of their own (artificial body). What the participants saw was also presented on the screen of a PC computer (Dell Precision Tower 5810, Steam VR software) placed behind them. This allowed the experimenters to check that the participants were continuously looking at their arm (artificial arm). Another computer was used (Vaio Sony) to present the experimental events followed by the two experimenters and to record the participants’ verbal responses. The timing of events was controlled via E-prime II software, thus making it possible to ensure that the visual and tactile stimulations were synchronous and that there was no delay that was noticeable to the participants. On the computer screen, two arrows of different colors, one color for each experimenter, warned them when to start (downward arrow) and finish (upward arrow) to stroke the participant and the mannequin. These arrows were also used in the test phase to warn when the experimenter 2 should delivered the first and the second stimulation on the mannequin’s arm indicating the onset and the offset of temporal interval to be judged. The experimenters were given training beforehand to manage experimental events and to enable them to repeat the movement identically, using the same force, speed, and velocity (for a similar procedure, see Ehrsson, 2007).

### Procedure

Throughout the entire experiment, the participants viewed the mannequin’s arm as if it were their own. They were assigned to two experimental conditions: a condition in which a body illusion was induced (synchronous condition) and a control condition (asynchronous condition). The condition order was counterbalanced within subjects. To induce the body illusion, the participants were given synchronized visual and tactile stimuli in the form of stroking. Experimenter 1 stroked the participant’s arm (out of view) in synchrony with experimenter 2, who stroked the mannequin’s arm (in full view) (Fig. 1). The visual information that the participants saw on the artificial body thus corresponded to the tactile stimulation felt on their own bodies. In the control condition, the visual and tactile stimulations were asynchronous. Experimenter 1 stroked the participant’s arm after experimenter 2 had stroked the mannequin’s arm. In each condition, the synchronous and asynchronous stroking movement was repeated 30 times.

Immediately after each condition (30 synchronous or 30 asynchronous strokes), the participants performed two test trials. In these two trials, they did not receive any tactile stimulation. They only saw two touches (simple quick touch) given by the experimenter 2 with the pen on the mannequin’s arm, that were separated by a temporal interval. The first touch on the first mark on the mannequin’s arm and the second on the second mark thus indicated the onset and the offset, respectively, of the temporal interval to be estimated. The task of the participant was therefore to judge the interval duration between the two stimuli perceived on the mannequin’s arm. The inter-stimulus interval duration was randomly chosen between 4 and 8 s. The participants verbally reported their temporal judgment. They were told not to count because this might bias the scientific results (Rattat & Droit-Volet, 2012). After each temporal judgment, the participants also reported their out-of-body experience (“I felt the effect of this stimulation in my own body”) on a 9-point scale going from “not at all” to “entirely (or absolutely)”.

During the temporal interval, the participants also saw a pleasant stimulus or a threatening stimulus. The pleasant/unpleasant trial order was chosen at random. The pleasant stimulus was a 1-s touch on the artificial arm with the soft paintbrush and the unpleasant one the same touch with the knife. Through their glasses, the participants therefore saw experimenter 2’s hand holding the soft paintbrush or the knife, making a quick movement and touching the mannequin’s arm. A preliminary study conducted on 23 other participants to test the stimuli used in the present study confirmed that the trials with the unpleasant stimulus were judged less pleasant (*M* = 3.48, *SE* = 0.286) than those conducted with the pleasant stimulus (*M* = 5.16, *SE* = 0.286), *E* =  − 1.68, *SE* = 0.139, 95% CI [−1.95 to −1.41], *t* =  − 12.1, *p* < .001, on a 9-point verbal scale going from not pleasant to very pleasant. The trials also tended to be judged more pleasant in the synchronous condition (*M* = 4.52, *SE* = 0.287) than in the asynchronous condition (*M* = 4.08, *SE* = 0.287), *E* = 0.432, *SE* = 0.157, 95% CI [0.122–0.74], *t* = 2.74, *p* = .006. Indeed, the participants reported finding the out-of-body experience more pleasurable when they felt the stimulation on their bodies, *E* = 0.125, *SE* = 0.456, 95% CI [0.355–0.215], *t* = 2.74, *p* = .006. Therefore, when the participants saw the mannequin’s arm in a first-person perspective, this did not generate a particularly pleasant experience, as indicated by the mean score of 4.298 on the 9-point scale. However, the synchronization condition was judged to be a more pleasant experience.

Each participant performed the experiment three times in the synchronous and the asynchronous conditions (for each, 30 strokes followed by the two temporal trials (pleasant/unpleasant)). As reported above, the condition order was counterbalanced within subjects. This resulted in a total of 12 test trials, i.e., 6 trials for each condition (2 × 6): 3 trials with the pleasant and with the unpleasant stimulus.

### Statistical analyses

**Figure 2:**
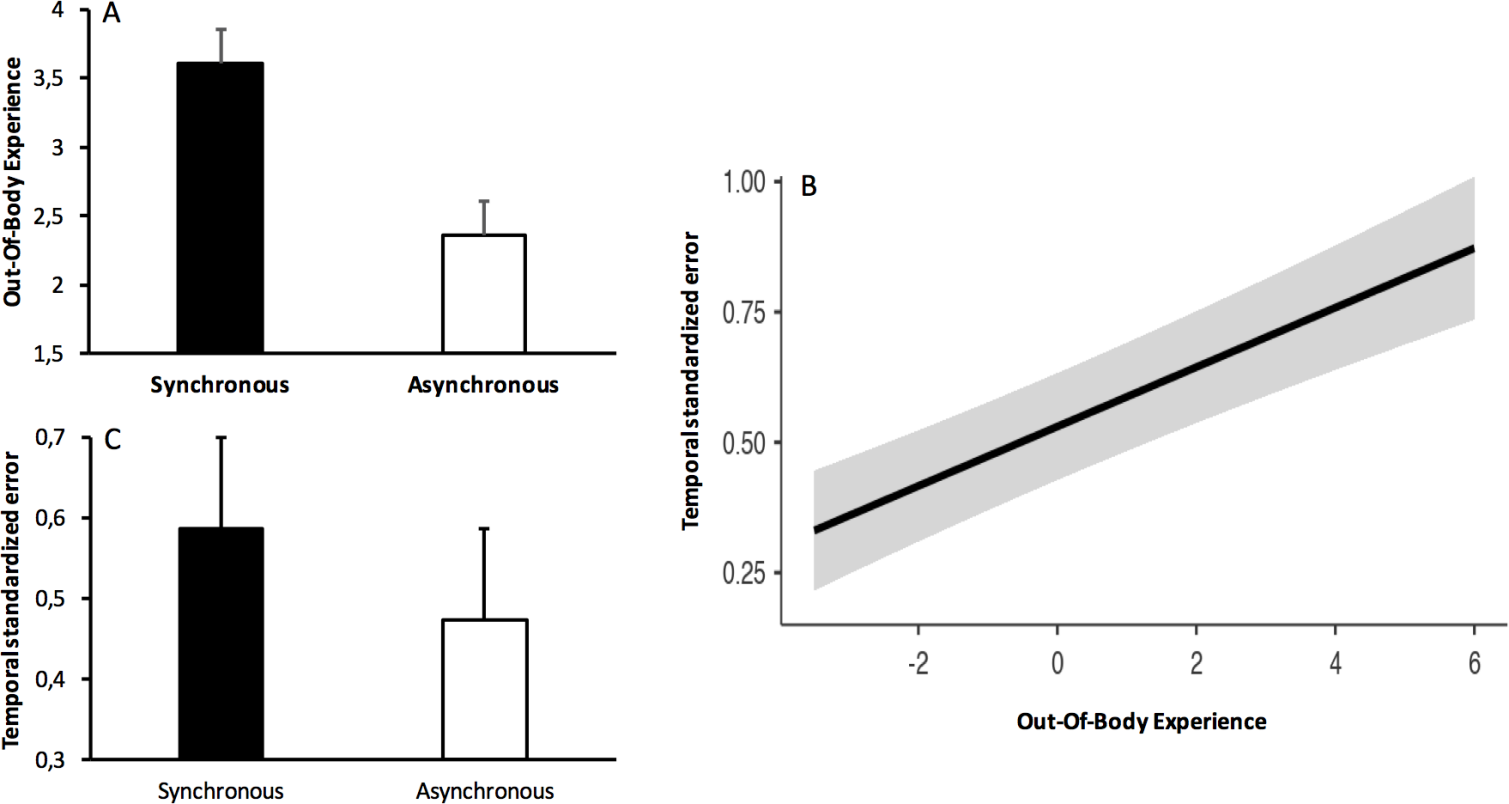
(A) Mean and standard error of out-of-body experience scores in the synchronous and the asynchronous condition. (B) Time judgment plotted against out-of-body experience scores. (C) Mean and standard of time judgment in the synchronous and the asynchronous condition.

The duration interval changed from one to another trial because it was randomly selected between 4 and 8 s. We thus calculated a temporal standardized error, i.e., the difference between the temporal estimate and the interval duration divided by the interval duration. A standardized error greater than zero indicates a temporal overestimation and one smaller than zero a temporal underestimation. In our study, the participants thus tended to overestimate durations in all experimental conditions (Fig. 2). In addition, as there were multiple non-independent responses for the same subject for each trial (temporal verbal judgment, out-of-body feeling), a linear mixed model (LMM) was used with the temporal standardized error as dependent variable and the synchronous/asynchronous condition, the pleasant/unpleasant condition or the out-of-body experience used as fixed factor, with the participants and the trials as random variables. The model was run using the jamovi software (2019).

## Results

Figure 2A shows the participants’ feeling, in their own bodies, of the stimulation administered to the mannequin after the synchronous or the asynchronous touching condition. The participants reported feeling the stimulation in their own bodies more in the synchronous (*M* = 3.61, *SE* = 0.243) than in the asynchronous condition (*M* = 2.36, *SE* = 0.243), *E* =  − 1.25, *SE* = 0.125, 95% CI [−1.49 to −1.01], *t* =  − 10.00, *p* < .001. The emotional valence of the stimuli (pleasant–unpleasant) did not modify this out-of-body experience (*p* > .10). Analyses on the first trial of the full trial series did not change this result.

As illustrated in Fig. 2B, the out-of-body experience was a significant predictor of time judgments: The more subjects embodied what they saw being administered to the mannequin’s body, the more time was overestimated, *E* = 0.531, *SE* = 0.0149, 95% CI [0.0285–0.087], *t* = 3.87, *p* < .001. The interval duration was indeed judged longer in the synchronous (*M* = 0.584, *SE* = 0.107) than in the asynchronous condition (*M* = 0.471, *SE* = 0.107), *E* =  − 0.113, *SE* = 0.0492, 95% CI [−0.209 to −0.016], *t* =  − 2.87, *p* < .02 (Fig. 2C). The time judgment did not change with the valence of the stimulation (*p* > .10). Finally, when all fixed factors were included in the same equation, only the out-of-body experience remained a reliable predictor of time judgments (*p* < .001), with both the experimental condition and the valence of the stimuli being non-significant (both *p* > .10). In conclusion, the time judgment was modified when the participants experienced the artificial body as belonging to the self, even in the absence of a tactile sensation on their own physical body.

## Discussion

The results of our study using the out-of-body paradigm demonstrated that the synchronous stroking of the participant’s invisible arm and the mannequin’s visible arm induced the illusion that the mannequin’s arm was a part of the participant’s own body. This illusion was weak with the asynchrony between the visual and tactile stimulation, even when the artificial arm was seen from a first-person perspective. Indeed, although the participants only saw the tactile stimulation of the mannequin’s arm in the test trials, they felt these tactile stimulations in their own bodies more strongly after the synchronous than the asynchronous stroking condition. Our results therefore replicate the findings of studies on the out-of-body experience, showing that the source of bodily self-identification lies in the matching between visual and somatosensory information (Ehrsson, 2007; Schmalzl & Ehrsson, 2011; Petkova & Ehrsson, 2001). They also confirm the robustness of the out-of-body illusion (Schmalzl & Ehrsson, 2011).

The originality of our results was to show that the duration judgments were related to the degree to which the stimulation of the artificial body was felt in the participants’ own bodies. The duration interval was indeed judged longer in the synchronous than in the asynchronous stroking condition. The significant link observed in our study between the time judgment and the feeling of stimulation in the participants’ own bodies, without direct tactile information, demonstrates the key role of the embodiment of stimulation in the judgment of durations. According to Ehrsson (2007, p. 1048), “the natural in-body experience forms the foundation of self consciousness”. Therefore, our results suggest that the judgment of time is modulated by self-awareness and the associated experiences. This is consistent with the results of recent studies reported in our introduction suggesting the critical role of the self in the explicit judgment of duration and the judgment of the passage of time (e.g., Droit-Volet & Dambrun, 2019; Wittmann, 2018). This also allows us to better understand difficulties in judging duration and temporal order in patients with schizophrenia suffering from a disorder of body-self awareness (e.g., Giersch, Lalanne & Isope, 2016).

However, our results did not show any significant difference in time judgments between the pleasant and unpleasant stimuli used in our study, even in the synchronous condition. In a preliminary study, we tested another unpleasant and pleasant stimulation, namely stroking over a distance of 15 cm with either a soft paintbrush or a pointed wooden stick. This approach was inspired by the procedure used by Ogden et al. (2015) to examine the effect of pleasant and unpleasant tactile stimulations on time judgment. However, the results were similar, with the valence of the tactile stimulation having no significant effect on time judgment. This is why we decided to use a more threatening stimulus (knife) as in the out-of-body experiments conducted on the rubber hand. Nevertheless, as the results of the current study show, we did not find any significant temporal effect of valence even with the new stimuli. One possible explanation is that the difference between the feelings of pleasure and displeasure was not great enough to induce timing differences in the case of a tactile stimulation perceived on another body. In a further study, it would be interesting to record an objective index of emotional reaction (e.g., skin-conductance response) in order to investigate whether an emotional reaction occurs despite the lack of distortion in time judgments. However, most studies on the out-of-body illusion have only used self-reported questionnaires (Ehrsson, 2007; Schmalzl & Ehrsson, 2011; Petkova & Ehrsson, 2001), and when objective indexes have been recorded, they have been found to be correlated with self-reported ownership (Ehrsson, 2007). Using functional magnetic resonance imaging (fMRI), Ehrsson, Spence & Passingham (2004) also showed a linear relationship between the self-reported rating of the illusion and the level of neural activity in the premotor cortex, which appears to be the neural mechanism responsible for bodily self-attribution. This validates the questionnaire data obtained for the out-of-body illusion. In our study, the participants thus reported a stronger illusion in the synchronous condition than in the control condition, but no difference in the bodily feeling as a function of the valence of the tactile stimulation provided during the inter-touch interval.

This study is the first to provide empirical data on time judgments in the out-of-body paradigm. Further experiments must now be conducted to try to automatize stimulation on the mannequin’s arm and to include, for example, the recording of a physiological index (electrodermal responses). Further experiments must also be conducted to better explain the temporal overestimation observed in our study. One might have assumed that, at the level of the minimal self, the awareness of the self in another body would have competed for cognitive resources required for other representations, in particular the processing of time. The participants should have then focused their attention on their own thoughts about the self to the detriment of attention allocated to time. However, if this were the case, we would have found an underestimation of time rather than a temporal overestimation, as was observed in our study. Indeed, most studies report an underestimation of interval durations when attention is distracted away from the processing of time. In addition, Gil & Droit-Volet (2011) showed that the perception of the emotion of self-consciousness (ashamed faces) produces a shortening rather than a lengthening of time estimates, which is caused by a reflection about the self and the perception of guilt (Lewis, 2007).

By contrast, as discussed in the introduction, the perception of bodily or facial expressions associated with highly arousing emotions has been systematically found to produce a lengthening effect, such as that observed in our study (for a review see Droit-Volet et al., 2013; Lake, 2016). A series of findings have demonstrated that the embodiment of an emotion perceived in a counterpart speeds up the perceiver’s internal clock system which provides the raw material (oscillations, pulses) for time measurement (e.g., Fayolle, Gil & Droit-Volet, 2015; Ogden et al., 2019). Consequently, more temporal units are recorded and the duration is judged longer. We can thus suggest that the overestimation of interval durations in the synchronous condition compared to the asynchronous condition may result from the embodiment of tactile stimuli perceived on the mannequin. An alternative hypothesis is that the temporal overestimation observed in the synchronous condition is only caused by the stress (emotion) induced by the out-of-body situation. In other words, this unusual experimental situation could have increased the arousal level which in itself could explain the results. It is difficult to respond without a physiological index of arousal level in our behavioral experiment. Further experiments are needed to test the role of emotion in our paradigm. Nevertheless, our results showed that the time judgment did not change with the type of emotional stimulation provided during the inter-touch interval duration (knife vs. paintbrush). In addition, preliminary data presented in the procedure revealed that the experimental situation was judged more pleasant in the synchronous condition than in the asynchronous condition, even if they saw an unpleasant emotional stimulus. Moreover, lengthening effects on timing have been found for unpleasant rather than for pleasant emotional stimuli in the studies on emotion (Droit-Volet et al., 2013). The relative pleasure in the synchronous condition is probably due to the congruence between the visual and tactile information that is more pleasant to live and less costly in terms of cognitive load than the incongruence between these pieces of information in the asynchronous condition. Sensori-motor synchronicity could thus induce out-of-body experience but also emotion, the two not necessary being linked. Nevertheless, our results support the assumption of an embodiment of time in the out-of-body illusion, or, in other words, that body experience, even if it is outside the body, affects the experience of time.

In the case of highly arousing emotions, the speeding up of the internal clock system results from the activation of the nervous system which automatically prepares the body for action. In an fMRI study, Ehrsson, Spence & Passingham (2004) observed the activation of a complex neural circuit (intraparietal cortex, dorsal premotor cortex, supplementary motor area, cerebellum, putamen, and thalamus) in the out-of-body task, but suggested that the premotor cortex is specifically involved in body-self attribution. Similarly, fMRI studies on timing have observed the activation of motor areas (supplementary motor area) in the explicit judgment of durations (Merchant, Harrington & Meck, 2013; Coull et al., 2015). There is therefore a converging set of data suggesting that the body and action play a key role in the conscious time judgments (Coull & Droit-Volet, 2018). Our study therefore provides additional data suggesting that body-self awareness and its associated sensory-motor experiences play a critical role in the subjective sense of time.

## Conclusion

The present study using the out-of-body illusion paradigm revealed the significant relationship between the out-of-body experience and the judgment of time. It thus highlights the importance of the awareness of the body-self in the processing of time, i.e., embodied time. However, further experiments are needed to better understand the temporal overestimation observed in our study on time and out-of-body.

##  Supplemental Information

10.7717/peerj.8565/supp-1 Supplemental Information 1 Raw data used in statistical analyses

## Additional Information and Declarations

### Data Availability

The following information was supplied regarding data availability: The raw measurements are available in the Supplemental Files.
